# P28. Efficient CD19-positive leukaemia cell lysis mediated by a T cell-recruiting triple body a[19-3-19]

**DOI:** 10.1186/2051-1426-2-S2-P19

**Published:** 2014-03-12

**Authors:** CC Roskopf, TA Braciak, C Schiller, S Wildenhain, N Fenn, U Jacob, GH Fey, KP Hopfner, FS Oduncu

**Affiliations:** 1 Klinikum der Universitaet Muenchen, Hemato-/Oncology MED IV, Munich, Germany; 2 Ludwig-Maximilians-Universitaet Muenchen, Gene Center, Munich, Germany; 3 Spectramab GmbH, Munich, Germany

## Background

The design of antibody derivatives with higher tumour cell selectivity is hoped to improve cancer immunotherapy and to decrease side effects. One promising molecular format that we have developed is the triple body. A triple body allows for selective lysis based on its 'dual targeting' capacity. The triple body format consists of three linked single chain variable fragments (scFv) with the two distal scFvs targeting different antigens on the tumour cell surface. The central scFv triggers an available immune effector cell for redirected lysis of the tumour cell. For the triple body anti[HLA-DR-CD16-CD19], it was previously shown that double-positive target cells were preferentially eliminated by NK cells versus single-positive targets. In the present study, a triple body designated α[19-3-19] with specificity for CD19 and CD3ε was constructed, produced and tested to determine whether a triple body could also recruit effector T cells as this has not previously been demonstrated.

## Materials and methods

For the construction and production of α[19-3-19], standard molecular biology procedures were employed. Flow cytometry was used to determine the specific binding behaviour of α[19-3-19] and to characterise various T cell subsets. The cytotoxic potential of α[19-3-19] was evaluated in redirected lysis assays using variable effector-to-target ratios against the B cell lines SEM, Nalm-6, Raji, Namalwa and ARH77 as well as cells from an MPAL patient.

## Results

We show that the triple body α[19-3-19] binds to CD19-positive cells and primary T cells. When tested in cytotoxicity assays, the triple body mediated up to 95% specific lysis of CD19-positive tumour cells from established B-ALL cell lines as well as against cells isolated from primary patient material using pre-stimulated allogeneic effector T cells *in vitro* after 3 hours incubation. At effector-to-target ratios of 1:10, α[19-3-19] still led to the depletion of approximately 60% of CD19-positive cells. Furthermore, the α[19-3-19] triplebody activated resting T cells from healthy unrelated donors and induced the specific lysis of autologous CD19-positive cells.

## Conclusion

These results show that the molecular format of the triple body using CD3ε as a T cell trigger is also suitable for the specific killing of leukaemia cells. In addition, the 'dual-targeting' capacity of triple bodies could make these molecules potentially powerful therapeutic agents for the selective and efficient elimination of leukaemia cells while sparing normal healthy cells.

